# Early Antiretroviral Therapy in AIDS Patients Presenting With *Toxoplasma gondii* Encephalitis Is Associated With More Sequelae but Not Increased Mortality

**DOI:** 10.3389/fmed.2022.759091

**Published:** 2022-02-25

**Authors:** Nadia Cubas-Vega, Paola López Del-Tejo, Djane C. Baia-da-Silva, Vanderson Souza Sampaio, Bruno Araújo Jardim, Monique Freire Santana, Luiz Carlos Lima Ferreira, Izabella Picinin Safe, Márcia A. Araújo Alexandre, Marcus Vinícius Guimarães Lacerda, Wuelton Marcelo Monteiro, Fernando Val

**Affiliations:** ^1^Programa de Pós-Graduação em Medicina Tropical, Universidade do Estado do Amazonas, Manaus, Brazil; ^2^Instituto de Pesquisa Clínica Carlos Borborema, Fundação de Medicina Tropical Dr. Heitor Vieira Dourado, Manaus, Brazil; ^3^Instituto Leônidas and Maria Deane, Fiocruz-Amazonas, Manaus, Brazil; ^4^Gerência de Endemias, Fundação de Vigilância em Saúde do Amazonas, Manaus, Brazil; ^5^Programa de Pós-Graduação em Ciências da Saúde, Universidade Federal do Amazonas, Manaus, Brazil; ^6^Departameto de Patologia e Medicina Legal, Universidade Federal do Amazonas, Manaus, Brazil; ^7^Departamento Clínico, Fundação de Medicina Tropical Dr. Heitor Vieira Dourado, Manaus, Brazil

**Keywords:** *Toxoplasma gondii*, toxoplasmic encephalitis, HIV/AIDS, therapy, antiretroviral, complications

## Abstract

**Background:**

Evidence on the optimal time to initiate antiretroviral therapy (ART) in the presence of toxoplasmic encephalitis (TE) is scarce. We compared the impact of early vs. delayed ART initiation on mortality and neurologic complications at discharge in a Brazilian population co-infected with HIV and TE.

**Methods:**

We retrospectively evaluated data from 9 years of hospitalizations at a referral center in Manaus, Amazonas. All ART-naïve hospitalized patients were divided into early initiation treatment (EIT) (0-4 weeks) and delayed initiation treatment (DIT) (>4 weeks). The groups were compared using chi-square test and mortality at 16 weeks.

**Results:**

Four hundred sixty nine patients were included, of whom 357 (76.1%) belonged to the EIT group. The median CD4^+^ lymphocyte count and CD4^+^/CD8^+^ ratio were 53 cells/mm^3^ and 0.09, respectively. Mortality rate and presence of sequelae were 4.9% (*n* = 23) and 41.6% (*n* = 195), respectively. Mortality was similar between groups (*p* = 0.18), although the EIT group had the highest prevalence of sequelae at discharge (*p* = 0.04). The hazard ratio for death at 16 weeks with DIT was 2.3 (*p* = 0.18). The necessity for intensive care unit admission, mechanical ventilation, and cardiopulmonary resuscitation were similar between groups.

**Conclusion:**

In patients with AIDS and TE, early ART initiation might have a detrimental influence on the occurrence of sequelae.

## Introduction

Toxoplasmic encephalitis (TE), caused by the intracellular protozoan parasite *Toxoplasma gondii*, is the most common central nervous system (CNS) opportunistic infection (OI) of people living with the human immunodeficiency virus (PLHIV) (1–3). If left untreated, it is a life-threatening condition that may cause different cognitive, psychological, and physical sequelae (4, 5). In Brazil, where clinical management of HIV/AIDS is provided free of charge (6), and despite the fact that the advent of highly active antiretroviral therapy (ART) has significantly decreased the morbidity and mortality of HIV/AIDS (7–9) in the past years, the prevalence of TE is a major cause of hospitalization in PLHIV and still represents a challenge for the healthcare system (10, 11). The treatment of patients with HIV and acute neurological infections (ANI) is challenging and the optimal timing to initiate ART remains a topic of major discussion (12–14).

The Brazilian Ministry of Health recommends the early initiation of ART in all PLHIV, regardless of symptoms and CD4^+^ T cell count, except for those with active meningeal tuberculosis and cryptococcal meningoencephalitis. In these patients, ART should be postponed 4 and 6 weeks after the initiation of tuberculous and fungal treatment, respectively (6). Similar approaches to the initiation of ART in PLHIV can be found in recommendations provided by international panels of experts (15, 16). Studies and data on when to start ART for PLHIV with TE and its impact are scarce. In this study, we aimed to compare major clinical outcomes of early vs. delayed ART initiation time in ART-naïve patients with HIV-associated TE who were admitted to an infectious disease referral center in the Amazonas state, Western Brazilian Amazon.

## Materials and Methods

### Study Design and Participants

This study collected retrospective data from HIV-infected patients with a clinical/radiological diagnosis of TE admitted to the Fundação de Medicina Tropical Dr Heitor Vieira Dourado (FMT-HVD) between January 2010 and December 2018. FMT-HVD is a tertiary-care referral hospital for infectious diseases located in Manaus, Western Brazilian Amazon, which receives patients seeking medical care as well as those referred from public and private health care units in surrounding localities. FMT-HVD is part of the Brazilian public health network and adopts all Brazilian guidelines for the management of sexually transmitted infections (17) and HIV infection (6). Clinical assistance, diagnostic tests, treatment, and follow-up are free of charge to all Brazilians and foreigners.

**Figure 1:**
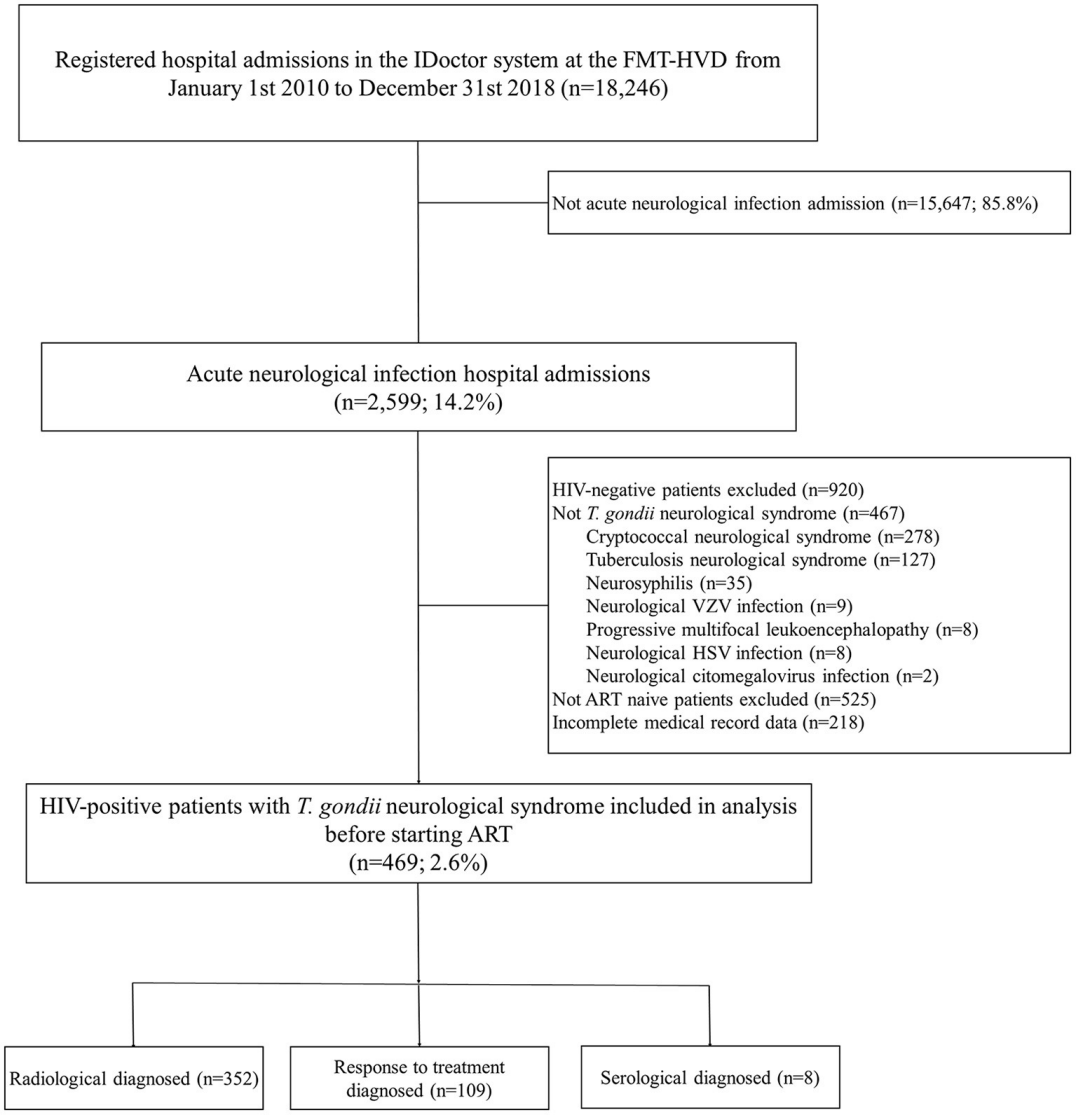
Study flowchart of inclusion of ART-naïve hospitalized patients co-infected with HIV and *T. gondii* encephalitis.

All patients are registered in the hospital's electronic medical record (EMR), the IDoctor platform, from which data were collected after a thorough screening process. Data on demographics, clinical, radiological and/or laboratory diagnosis of TE, length of hospitalization and any supportive procedures [intensive care unit (ICU), mechanical ventilation (MV), cardiopulmonary resuscitation (CPR)] and outcome (alive or dead) were retrieved from individual EMRs and sent anonymously to the databank. Selection of eligible participants for analysis is shown in Figure 1.

HIV infection was determined by two different consecutive positive 3rd generation rapid tests (RTs, Abon Alere [Abbott, Chicago, United States] and Bio-Manguinhos [Fiocruz, Rio de Janeiro, Brazil]), and confirmed by an immunoassay test, as defined by the Brazilian Ministry of Health (18). TE was defined by a clinically compatible presentation, most frequently described as headache, focal neurological deficit, fever, mental confusion and seizures (11, 19), either alone (after exclusion of other causes) nor in addition to computerized tomography (CT) or magnetic resonance imaging (MRI) compatible findings (enhancing brain lesions with perilesional edema, with or without expansive effect), serology for Anti-*T. gondii* IgG and/or IgM antibodies, and a satisfactory response after specific treatment (pyrimethamine, sulfadiazine, and folinic acid, or any other recommended by national guidelines) (6).

Only new episodes of TE were registered. If a patient had other hospitalizations registered in the database due to the same cause, they were considered a duplicate, and excluded. The first laboratory data (hematology and biochemistry) after admission and either the first CD4^+^, CD8^+^, and viral loads counts after admission (newly diagnosed HIV infection) or the most recent pre-admission CD4^+^, CD8^+^, and viral loads counts (people already living with HIV at the time of TE diagnosis) were recorded. Data collection was carried out by two independent researchers. Any disagreements were resolved by consensus.

### Outcome Assessments

The included patients were divided into groups according to the ART initiation time as described elsewhere (14). Briefly, early initiation treatment (EIT) and delayed initiation treatment (DIT) referred to ART starting within the first 4 weeks (28 days) or after 4 weeks (>28 days) of TE diagnosis, respectively. Only ART-naïve patients who had available information regarding ART initiation time on the EMRs were included in this study (Figure 1). Early or delayed initiation of treatment followed the physician's discretion. Deciding which antiretroviral regimen to use was guided by the local protocols in use at the time (6, 20, 21).

The primary study outcome was in-hospital death. Secondary outcomes were the presence of sequelae at hospital discharge, need for ICU, MV, CPR, and number of hospitalization days. Neurological sequelae recorded in the study were defined as the presence until medical discharge, of one or more of the baseline signs or symptoms or the development of a new one; and for their evaluation the last medical statement prior to discharge was reviewed. Patients were followed up until hospital discharge or time of in-hospital death.

### Statistical Analysis

Socio-demographic and clinical characteristics of patients were summarized as mean and standard deviation (SD) when distributions were confirmed as normal. Otherwise, data were expressed as medians and interquartile ranges (IQR). Categorical variables are reported as frequency and percentage. Comparative statistics between ART initiation groups were performed using Chi-square test for categorical variables and the Wilcoxon test or the Student's *t*-test for continuous variables. Shapiro-Wilk test was used to verify normality. Mortality at 16 weeks was compared between groups with the use of Peto-Peto-Prentince test and Cox-regression, which was performed to derive the hazard ratio (HR) two-sided 95% confidence intervals (CI) and represented with Kaplan-Meier curves. Each patient's survival time began on the date of hospitalization and ended on the date of death or hospital discharged. All data were analyzed using Stata® version 13 (StataCorp., College Station, Texas, United States). Statistical significance was set as a value of *p* < 0.05.

### Ethical Considerations

The study protocol was approved by the FMT-HVD Ethics Review Board (ERB) (approval number 3.085.269/2018). Due to the retrospective nature of data collection and analysis, the ERB granted a waiver of the requirement to obtain informed consent.

## Results

### Study Population

A total of 2,599 patients were diagnosed with an acute neurological infection during the study period. Of these, 469 (18.0%) were ART-naïve HIV-TE patients (Figure 1). HIV cases per year are shown in Supplementary Figure 1, along with a timeline describing the changes in ART outlined by the Brazilian Ministry of Health throughout the study period. Table 1 summarizes baseline characteristics and outcomes. Men (*n* = 339, 72.3%), with a median age of 35 years (IQR: 28-44) and living in an urban area (*n* = 443, 94.5%) were the most affected. Headache (*n* = 315, 67.2%), limb paresis (*n* = 295, 62.9%) and altered consciousness level (*n* = 230, 49.0%) were the most frequent neurological signs at hospitalization. Clinical presentation at admission and laboratory baseline data are shown in Supplementary Table 1. Fifty-eight (12.4%) individuals were aware of the HIV status, with the time interval between HIV diagnosis and neurological clinical manifestation ranging from 1 month to 11 years. A total of 357 (76.1%) individuals were EIT, with a median time between hospital admission and ART initiation of 10 days (IQR: 5–16 days); 112 (23.9%) were in the DIT group (median ART initiation time 62 days, IQR: 40-221).

**Table 1:** Baseline characteristics and outcome according to ART initiation time.

**Variables**	**Total (*n =* 469)**	**Early ART** **(*n =* 357)**	**Delayed ART (*n =* 112)**	**p-value**	**Completeness (%)**
**Demographics**					
Males *n* (%)	339 (72.3)	262 (73.4)	77 (68.8)	0.339	100
Age (years) median (IQR)	35 (28–44)	36 (28–45)	35 (29–43)	0.455	100
Residence in urban area *n* (%)	443 (94.5)	338 (94.7)	105 (93.8)	0.708	100
**HIV historical**					
Viral load (10^3^copies/mL) median (IQR)	130,640 (17,842–387,490)	143,069 (16,545–409,125)	90,044 (21,089–211,584)	0.275	67
CD4^+^ count (/mm^3^) median (IQR)	53 (25–104)	53 (25–105)	56 (30–89)	0.938	83
CD8^+^ count (/mm^3^) median (IQR)	597 (361–1,017)	592 (361–1,017)	654 (383–989)	0.418	74
CD4/CD8 ratio median (IQR)	0.090 (0.049–0.162)	0.090 (0.048–0.164)	0.085 (0.0510–0.155)	0.778	74
**Noninfectious comorbidities** ***n*** **(%)**	114 (24.3)	95 (26.6)	19 (17.0)	0.038	100
Hypertension *n* (%)	33 (7.0)	27 (7.6)	6 (5.4)	0.426	
Diabetes mellitus *n* (%)	14 (3.0)	12 (3.4)	2 (1.8)	0.393	
Neoplasia *n* (%)	3 (0.6)	3 (0.8)	0 (0.0)	0.330	
Other comorbidities^a^ *n* (%)	70 (14.9)	58 (16.2)	12 (10.7)	0.152	
**Coinfections**	310 (66.1)	242 (67.8)	68 (60.7)	0.168	100
Multiple neuroinfections^b^ *n* (%)	28 (6.0)	24 (6.7)	4 (3.6)	0.219	
Oropharyngeal candidiasis *n* (%)	182 (38.8)	140 (39.2)	42 (37.5)	0.745	
Respiratory tract infections^c^ *n* (%)	103 (22.0)	72 (20.2)	31 (27.7)	0.094	
Gastrointestinal infections *n* (%)	19 (4.1)	15 (4.2)	4 (3.6)	0.768	
Other concomitant infections^d^ *n* (%)	72 (15.4)	57 (16.0)	15 (13.4)	0.510	
**Outcomes**					
Death *n* (%)	23 (4.9)	17 (4.8)	6 (5.4)	0.799	100
Hospitalization days median (IQR)	19 (10–32)	20 (11–31)	18 (9–40)	0.574	100
Sequels^e^ *n* (%)	195 (41.6)	158 (44.3)	37 (33.0)	**0.036**	
ICU^f^ *n* (%)	37 (7.9)	27 (7.6)	10 (8.9)	0.640	100
MV^g^ *n* (%)	31 (6.6)	23 (6.4)	8 (7.1)	0.795	100
CPR^h^ *n* (%)	10 (2.1)	8 (2.2)	2 (1.8)	0.771	100

a *Other, gastrointestinal diseases, psychiatric disorders, dermatological diseases, heart diseases, kidney disorders, and among others*;

b *CNS tuberculosis (n = 11), cryptococcal meningitis (n = 8), neurosyphilis (n = 8), and Candida tropicalis (n = 1)*;

c *pulmonary tuberculosis included (n = 61)*;

d *Other, viral hepatitis, genitourinary infections, skin infections, and among others*;

e *Including limb paresis, persistent headache, communication disorder, limb paresthesia, seizure syndromes, cranial nerve involvement, uncoordinated movements, muscle spasticity, visual disturbances, plegia, pyramidal and extrapyramidal signs, tubes, cannulas or ostomies at discharge, and memory and cognitive deficit*;

f *ICU, intensive care unit*;

g *MV, mechanical ventilation*;

h *CPR, cardiorespiratory resuscitation. Bold values mean that they are statistically significant*.

### Diagnosis and Treatment

A total of 352 (75.0%) patients were diagnosed from radiological findings, 8 (1.7%) had positive IgM (7 with positive IgG), and 109 (23.3%) diagnosed according to clinical signs and symptoms and response to treatment (Figure 1). Of the total patients, 296 (63.1%) had information on serology for *T. gondii*, out of these, 290 (98.0%) had evidence of previous exposure to the disease with positive IgG quantitatively performed by chemiluminescent immunoassay (DiaSorin S.p.A., Saluggia, Italy).

Three hundred and ten (66.1%) patients presented different coinfections; among those, 28 (9.0%) were diagnosed by the treating physicians with more than one infectious agent responsible for the neurological infection, all of them with two simultaneous etiological agents (Table 1). Pyrimethamine, sulfadiazine, and folinic acid (*n* = 398, 84.9%) were the most common drugs used for TE treatment. In 151 (32.2%) individuals, corticosteroids were used as adjuvant therapy, without significant difference of distribution between groups (*p* = 0.80). A combination of nucleoside/nucleotide reverse transcriptase inhibitors (NRTIs) and non-nucleoside reverse transcriptase inhibitors (NNRTIs) were the most frequently used ART in both groups (EIT: *n* = 229, 64.1% and DIT: *n* = 76, 67.9%) (Supplementary Table 2), without significant association with the development of sequelae (*p* = 0.21) or progression to death (0.96). No statistical association was found between death rate and corticosteroid use (*p* = 0.24).

### Study Outcomes

**Figure 2:**
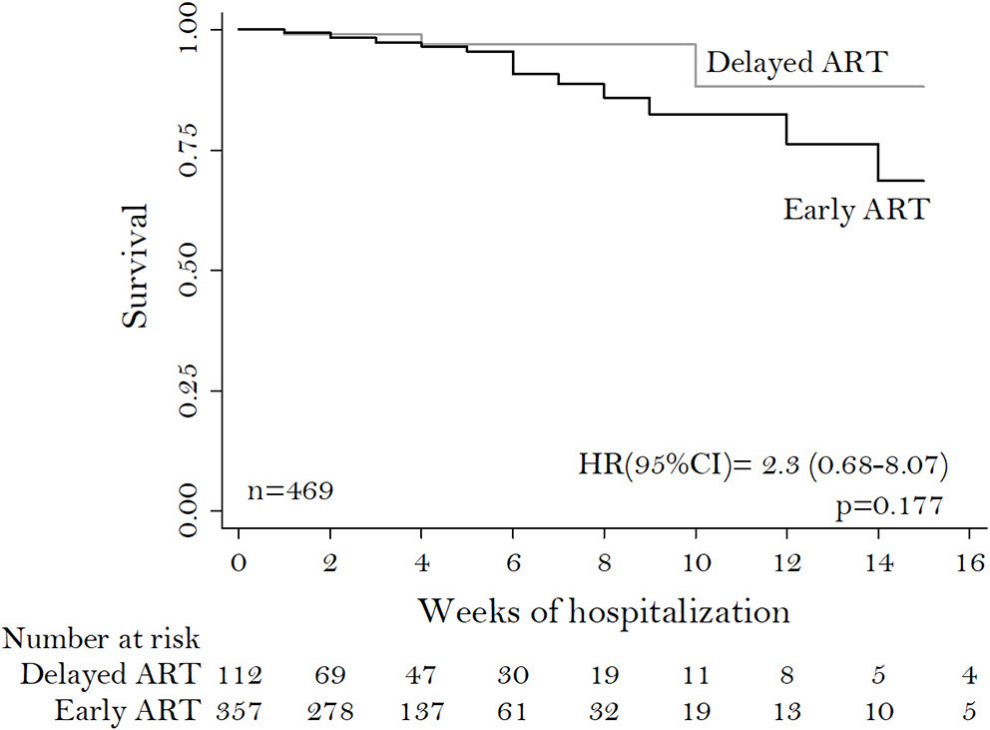
Kaplan-Meier curve of survival of ART-naïve hospitalized patients co-infected with HIV and *T. gondii* encephalitis.

The overall mortality was 4.9% (23/469), with higher mortality in the DIT group (5.4%, 6/112, Table 1), however, without statistical significance (*p* = 0.80). The overall sequelae rate was 41.6% (*n* = 195), with a higher prevalence of sequelae in the EIT group (158/357; 44.3%) compared to DIT (37/112, 33.0%), *p* = 0.04. The most prevalent sequelae at discharge were limb paresis (*n* = 105, 22.4%), followed by recurrent headaches with 11.1% (*n* = 52) (Supplementary Table 3). No statistical significance was found in the distribution for the need for ICU, MV, and CPR (Table 1). In general, the median length of survival time from admission was 28 weeks (IQR: 18–34). The HR for death at 16 weeks for the DIT group was 2.3 (95%CI 0.68–8.07; *p* = 0.17) (Figure 2).

## Discussion

Opportunistic infections (OIs) are the leading cause of mortality in the HIV population (22). Currently, the evidence to guide ART initiation time in PLHIV in low- and middle-income countries co-infected with OIs is limited and based on studies with different socioeconomic and demographic characteristics (23, 24), as also documented in this study. In these countries, scientific research is limited (25, 26) and management of infectious diseases is challenging (27, 28). To our knowledge, not many studies investigate the association between timing of ART initiation and mortality and prevalence of sequelae in hospitalized patients with HIV and TE, regardless of the potential for CNS infections to cause cognitive and/or motor sequelae (5, 29). Our findings indicate that mortality was similar in both groups, but the prevalence of sequelae was lower in those who started ART after 4 weeks, which suggests an association with a better clinical sequelae profile when starting ART in a delayed manner in this population.

The population in our study has three notable characteristics (i) ART-naïve HIV individuals, (ii) the occurrence of TE as an influential factor on the decision to start ART early or administer treatment for the coinfections first, and (iii) a large proportion of patients who at the time of inclusion in the study already had advanced HIV/AIDS disease, with low CD4^+^ lymphocyte count and CD4^+^/CD8^+^ ratio, and a high viral load. The presence of an ANI in ART-naive patients will always bring major therapeutic challenges associated with the possibility of developing immune reconstitution inflammatory syndrome (IRIS) (30, 31) and complex pharmacokinetic interactions and pharmacological co-toxicities (32, 33), which not only occur between ART and coinfections' treatment but also with drugs used chronically to treat other comorbidities (34, 35).

In several decades of the epidemics, Brazilian health authorities have been concerned with the quality of care offered to people with HIV/AIDS (36, 37). Of note, TE remains a persistent problem in the Amazon (38). Furthermore, the morbimortality due to AIDS continues to exceed the national rate (Amazonas: 6.4%, Brazil: 4.1%) (8). Until 2013, the Brazilian Ministry of Health recommended initiation of ART in symptomatic or asymptomatic individuals with CD4^+^ cells below 350 cells/mm^3^ (21). At the end of that year, treatment guidelines were updated to recommend ART in all symptomatic PLHIV regardless of the CD4^+^ count, and asymptomatic individuals with CD4^+^ <500 cells/mm^3^ (20). Currently, Brazilian HIV treatment guidelines recommends the start of ART in all PLHIV, despite clinical presentation and CD4^+^ counts (6). This Brazilian guidelines changes might have influenced the decision of when to start the ART in seven (2012–2018) out of the 9 years of this study period, as well as interfere in which antiretroviral drugs to use, since, as showed in the results, the combination of NRTI plus NNRTI was the most common choice in both groups.

Although the initiation of ART is associated with a lower mortality rate, HIV-infected individuals continue to present late for treatment and care, and consequently initiate ART with low CD4^+^ count and have a significantly higher risk of mortality (39, 40). The mortality rate in our series was 4.9%, which is lower than in a 20-year population based cohort study (31.0%) conducted in Denmark (5), where all TE patients had advanced HIV-disease and approximately 40% knew their serological status. Zolopa et al. (12), in a multicenter randomized trial with PLHIV and OIs, showed a decrease in mortality when ART was initiated within the first 2 weeks after diagnosis, compared to after 4 weeks. However, the study had a higher prevalence of patients with lung infections, not ART-naïve, and the whole population had CD4^+^ counts lower than 200 cells/mm^3^ and only 13 patients with HIV/TE were included. Similar findings were reported in a Spanish cohort study (13) involving ART-naïve patients with AIDS-defining diseases (with pulmonary infections being the most frequent), where late initiation of ART was associated with faster progression to death. In our study, the data reflect similar findings, in which patients with neurological infections due to *T. gondii*, who had ART introduced earlier, showed a trend toward lower mortality rates, although not statistically significant differences were demonstrated.

Sequelae can range from impairment of cranial nerves and headache to motor deficits and cognitive disorders (5, 29), occurring in up to 38% of patients (41). Here, sequelae at discharge were significantly more prevalent in the EIT group, indicating that the early start of ART in this population could be detrimental for this population. This could be due to the nature of the TE itself, because although, to our knowledge, no other study has directly addressed the relationship between the onset of ART and the incidence of sequelae, other studies have shown that patients coinfected with HIV-TE present more severe and persistent cognitive and motor deficits when compared with other ANI (23, 42, 43). It seems that early initiation of ART is always beneficial or does not interfere (44, 45), however the number of subjects in several of these studies is reduced in comparison to the present study. We show an elevated prevalence of sequelae, higher than the reported by Erdem et al. (46) (18.5%) regarding a multicenter study of CNS infections, with neurocryptococcosis and neurotuberculosis becoming the most prevalent diseases among 84 patients with HIV (41.6 and 18.5%, respectively). The same scenario is in agreement with the findings of an 11-year French study by Sonneville et al. (4) with PLHIV with TE admitted to the ICU, showing a lower prevalence of sequelae in their population (37.0%).

Our study has several limitations. Its retrospective nature accounts for incomplete data availability on patients' medical charts, mostly due to the absence of detailed targeted investigation, including proper registration of imaging and laboratory studies. The decision to admit patients and clinical management approaches were made at the physician's discretion, and may have resulted in classification bias for some of the outcomes here reported, which were minimized by the choice of hard clinical outcomes. Finally, our study could not fully characterize IRIS, associated with ART introduction, which has been shown to be associated with neurological sequelae and high mortality rates, especially in patients with cerebral tuberculosis and neurocryptococcosis (47, 48). Notwithstanding this, we do not believe that this is a major limitation, since several studies have reported a low incidence of IRIS in HIV-TE coinfection (49, 50), and the early or late introduction of ART does not seem to influence its onset (51, 52).

## Conclusions

This is the largest series of cases presenting data on mortality, prevalence of sequelae and secondary neurologic complications in people with HIV and TE. The analyzed data show that early ART initiation in this population might have a detrimental influence on the occurrence of sequelae in these patients. These findings have not been previously reported, as ART initiation in this population has been based on limited availability of clinical evidence (17), routinely within the first 2 weeks after diagnosis and TE treatment. Neurological infections remain a major challenge in Brazil, especially in increasing HIV prevalence scenarios and decreasing government interest in this population's health status. Further studies under controlled conditions to generate evidence are needed to corroborate these findings. Future research also needs to address the chronic complications of those who manage to survive ANI secondary to HIV/AIDS and OIs and provide insights into cognitive and physical disabilities and rehabilitation (53).

## Data Availability Statement

The raw data supporting the conclusions of this article will be made available by the authors, without undue reservation.

## Ethics Statement

The studies involving human participants were reviewed and approved by FMT-HVD Ethics Review Board. Written informed consent for participation was not required for this study in accordance with the national legislation and the institutional requirements.

## Author Contributions

NC-V, ML, WM, and FV: study concept and design. NC-V and PL: acquisition of the data. NC-V, VS, DB-d-S, and FV: analysis of the data. NC-V, DB-d-S, ML, and FV: drafting of the manuscript. NC-V, PL, DB-d-S, VS, BJ, MS, LF, IS, MA, ML, WM, and FV: critical revision of the manuscript and approval of final manuscript. All authors contributed to the article and approved the submitted version.

## Funding

This study was supported by the Coordenação de Aperfeiçoamento de Pessoal de Nivel Superior (CAPES) and the Fundação de Amparo à Pesquisa do Estado do Amazonas (FAPEAM) RESOLUÇÃO No. 002/2008, 007/2018 e 005/2019—Pró-Estado and RESOLUÇÃO No. 006/2020. WM and ML are CNPq (Conselho Nacional de Desenvolvimento Científico e Tecnológico) fellows. The funders had no role in study design, data collection, manuscript preparation, or decision to publish.

## Conflict of Interest

The authors declare that the research was conducted in the absence of any commercial or financial relationships that could be construed as a potential conflict of interest.

## Publisher's Note

All claims expressed in this article are solely those of the authors and do not necessarily represent those of their affiliated organizations, or those of the publisher, the editors and the reviewers. Any product that may be evaluated in this article, or claim that may be made by its manufacturer, is not guaranteed or endorsed by the publisher.

## Supplementary Material

Supplementary Figure 1 Distribution per year of ART-naïve hospitalized patients co-infected with HIV and *T. gondii* encephalitis and ART transition timeline during the study period.
