# Supplementary material for: Early Antiretroviral Therapy in AIDS Patients Presenting With Toxoplasma gondii Encephalitis Is Associated With More Sequelae but Not Increased Mortality
Source: Front Med (Lausanne). 2022 Feb 25;9:759091. doi: 10.3389/fmed.2022.759091 (PMC8914028; doi:10.3389/fmed.2022.759091)
Supplement: Supplementary file 1 [file Data_Sheet_1.docx]

| **Supplementary table 1.**  Clinical and laboratory baseline data from hospitalized ART-naïve hospitalized patients co-infected with HIV and *T. gondii* neurological syndrome. | |
| --- | --- |
| **Variables** | **Total**  **(n=469)** |
| **Signs and symptoms at hospital admission** |  |
| Headache n (%) | 315 (67.2) |
| Limb paresis n (%) | 295 (62.9) |
| Fever n (%) | 248 (52.9) |
| Mental confusion n (%) | 230 (49.0) |
| Weight Loss n (%) | 225 (48.0) |
| Vomiting n (%) | 153 (32.6) |
| Diarrhea n (%) | 149 (31.8) |
| Seizures n (%) | 140 (29.9) |
| Communication disorder n (%) | 119 (25.4) |
| Cranial nerve involvement n (%) | 115 (24.5) |
| Asthenia n (%) | 105 (22.4) |
| Cough n (%) | 96 (20.5) |
| Visual disturbances n (%) | 79 (16.8) |
| Limb paresthesia n (%) | 79 (16.8) |
| Nausea n (%) | 76 (16.2) |
| Altered behavior status n (%) | 52 (11.1) |
| Plegia n (%) | 53 (11.3) |
| Uncoordinated movements n(%) | 39 (8.3) |
| Muscle Spasticity n (%) | 19 (4.1) |
| Hearing disturbances n (%) | 8 (1.7) |
| Meningism signs n (%) | 3 (0.6) |
| **Complete blood count**^a^ **and blood chemistry tests** |  |
| Hemoglobin (g/dL) median (IQR) | 12 (10-13) |
| White blood cells (10^3^/mm^3^) median (IQR) | 5040 (3750-6860) |
| Platelets (10^9^/L) median (IQR) | 243,000 (187,000-317,000) |
| Neutrophils (10^3^/mm^3^) median (IQR) | 3,302 (2,248-4,777) |
| Eosinophils (10^3^/mm^3^) median (IQR) | 95 (50-188) |
| Lymphocytes (10^3^/mm^3^) median (IQR) | 1,060 (711-1,485) |
| Monocytes (10^3^/mm^3^) median (IQR) | 307 (191-471) |
| Glucose (mg/dL) median (IQR)^b^ | 106 (91-122) |
| Creatinine (mg/dL) median (IQR)^c^ | 0.8 (0.7-1.0) |
| Serum sodium mmol/L median (IQR)^c^ | 138 (134-142) |
| Serum potassium mmol/L median (IQR)^c^ | 4.0 (3.7-4.3) |
| AST^d^ IU/L median (IQR)^e^ | 33 (23-54) |
| ALT^f^ IU/L median (IQR)^g^ | 34 (20-56) |
| **HIV/AIDS** |  |
| Viral load (10^3^copies/mL) median (IQR)^h^ | 130,640 (17,842-387,490) |
| CD4+ count (/mm3) median (IQR)^i^ | 53 (25-104) |
| CD8+ count (/mm3) median (IQR)^j^ | 597 (361-1017) |
| CD4/CD8 ratio | 0.090 (0.049-0.162) |
| **Cerebrospinal fluid analysis**^k^ |  |
| Glucose mg/100mL median (IQR) | 52 (44-60) |
| Protein mg/100mL median (IQR) | 80 (45-126) |
| Cell count/mm^3^ median (IQR) | 15 (3-36) |
| Major leukocytes type |  |
| Mononuclear leukocytes n (%) | 169 (98.3) |
| Polymorphonuclear leukocytes n (%) | 3 (1.7) |
| **Imaging studies** |  |
| Abnormal CT^l^ findings n (%)^m^ | 345 (73.6) |
| Abnormal MRI^n^ findings n (%)^o^ | 92 (19.6) |
| ^a^ completeness: 463/469; ^b^ completeness: 415/469; ^c^ completeness: 461/469;  ^d^ AST: Alanine transaminase; ^e^ completeness:425/469;  ^f^ ALT: Aspartate transaminase; ^g^ completeness: 427/469;  ^h^ completeness: 315/469;  ^i^ completeness: 390/469; ^j^ completeness: 346/469; ^k^ completeness: 202/469; ;  ^l^ CT: computerized tomography; ^m^ completeness: 355/469;  ^n^ MRI: magnetic resonance imaging; ^o^ completeness: 94/469. | |

| **Supplementary table 2.**  Pharmacological treatment used in hospitalized ART-naïve patients co-infected with HIV and *T. gondii* neurological syndrome (n=469). | | | | |  |
| --- | --- | --- | --- | --- | --- |
| **Variables** | **Total**  **n=469**  **n (%)** | **Early ART**  **n=357**  **n (%)** | **Delayed ART**  **n=112**  **n (%)** | |  |
| ***Toxoplasma gondii* treatment** |  |  | | |  |
| Pyrimethamine, sulfadiazine, and folinic acid | 398 (84.9) | 287 (80.4) | | 100 (89.3) |  |
| Pyrimethamine, clindamycin, and folinic acid | 63 (13.4) | 52 (14.6) | | 11 (9,8) |  |
| TMP-SMX^a^, and folinic acid | 5 (1.1) | 5 (1.4) | | 0 (--) |  |
| TMP-SMX alone | 2 (0.4) | 2 (0.6) | | 0 (--) |  |
| Pyrimethamine, TMP-SMX, and folinic acid | 1 (0.2) | 0 (--) | | 1 (0.9) |  |
| **Antiretroviral treatment** |  |  | | |  |
| NRTIs^b^ + NNRTIs^c^ | 305 (65.0) | 229 (64.1) | | 76 (67.9) |  |
| NRTIs + IN^d^ | 109 (23.2) | 97 (27.2) | | 12 (10.7) |  |
| NRTIs + PIs^e^ | 54 (11.5) | 31 (8.7) | | 23 (20.5) |  |
| NNRTIs | 1 (0.2) | 0 (--) | | 1 (0.9) |  |
| **Corticosteroids** | 151 (32.2) | 116 (32.5) | | 35 (31.3) |  |
| Dexamethasone | 138 (29.4) | 104 (29.1) | | 34 (30.4) |  |
| Prednisone | 9 (1.9) | 8 (2.2) | | 1 (0.9) |  |
| Methylprednisolone | 4 (0.9) | 4 (1.1) | | 0 (--) |  |
| ^a^ Trimethoprim and sulfamethoxazole; ^b^ Nucleoside/nucleotide reverse transcriptase inhibitors; ^c^ Non-nucleoside reverse transcriptase inhibitors; ^d^ Integrase inhibitors; ^e^ Protease inhibitors. | | | | |  |

| **Supplementary table 3.**  Sequelae at discharge in hospitalized ART-naïve patients co-infected with HIV and *T. gondii* neurological syndrome (n=469). | | | |
| --- | --- | --- | --- |
| **Variables** | **Total**  **n=469**  **n (%)** | **Early ART**  **n=357**  **n (%)** | **Delayed ART**  **n=112**  **n (%)** |
| Limb paresis | 105 (22.4) | 88 (24.6) | 17 (15.2) |
| Headache | 52 (11.1) | 39 (10.9) | 13 (11.6) |
| Communication disorder | 36 (7.7) | 30 (8.4) | 6 (5.4) |
| Limb paresthesia | 33 (7.0) | 25 (7.0) | 8 (7.1) |
| Seizure syndromes | 25 (5.3) | 18 (5.0) | 7 (6.2) |
| Cranial nerve involvement | 19 (4.1) | 18 (5.0) | 1 (0.9) |
| Uncoordinated movements | 14 (3.0) | 11 (3.1) | 3 (2.7) |
| Muscle spasticity | 12 (2.6) | 9 (2.5) | 3 (2.7) |
| Visual disturbances | 10 (2.1) | 9 (2.5) | 1 (0.9) |
| Plegia | 9 (1.9) | 8 (2.2) | 1 (0.9) |
| Pyramidal and extrapyramidal signs | 9 (1.9) | 7 (2.0) | 2 (1.8) |
| Tubes, cannulas or ostomies | 8 (1.7) | 7 (2.0) | 1 (0.9) |
| Memory déficit | 4 (0.9) | 4 (1.1) | 0 (0.0) |
| Cognitive déficit | 3 (0.6) | 3 (0.8) | 0 (0.0) |
|  | |  |  |
