# Supplementary figures and images for: Early Antiretroviral Therapy in AIDS Patients Presenting With Toxoplasma gondii Encephalitis Is Associated With More Sequelae but Not Increased Mortality
Source: Front Med (Lausanne). 2022 Feb 25;9:759091. doi: 10.3389/fmed.2022.759091 (PMC8914028; doi:10.3389/fmed.2022.759091)

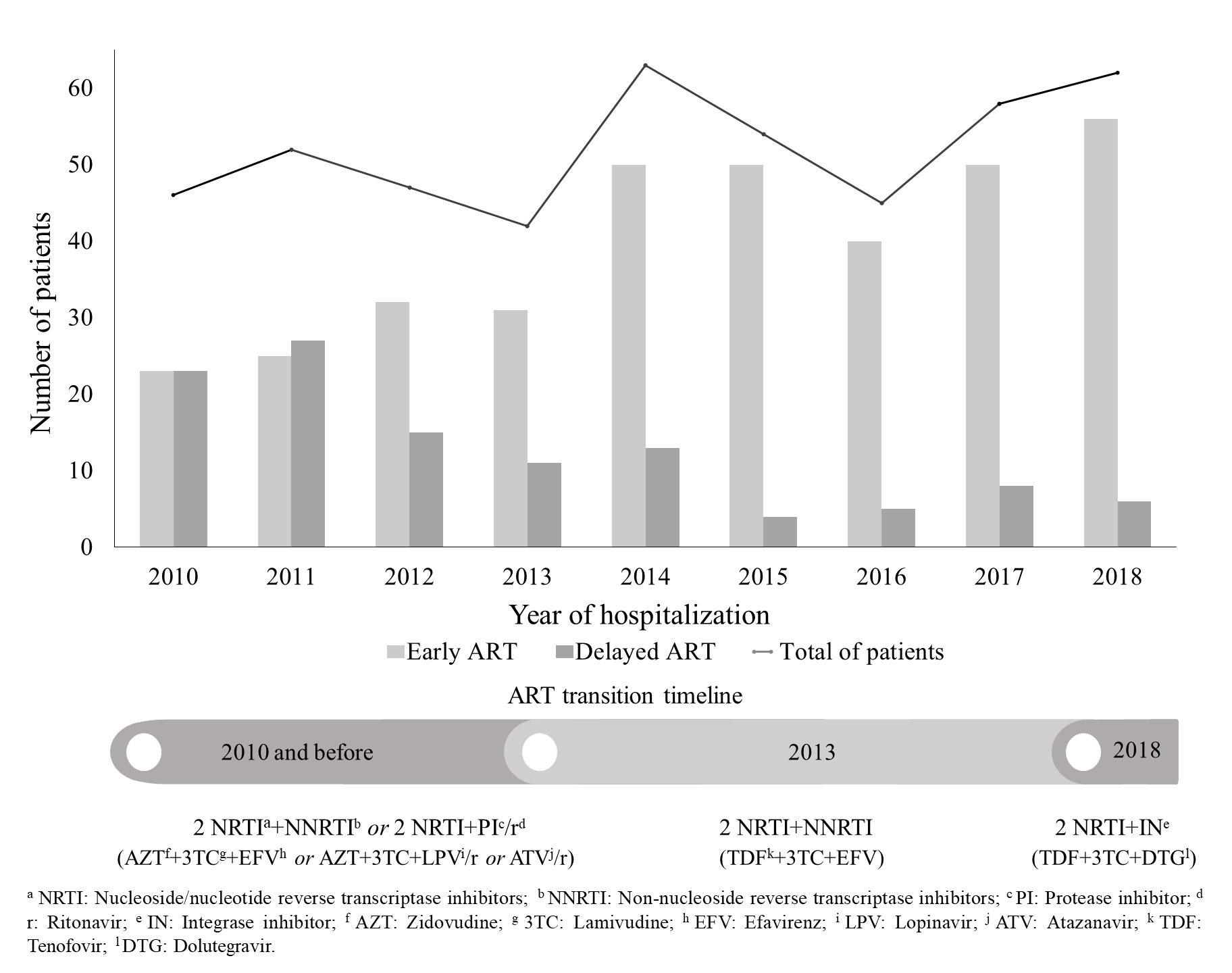

Supplement: Supplementary Figure 1 — Distribution per year of ART-naïve hospitalized patients co-infected with HIV and T. gondii encephalitis and ART transition timeline during the study period. [file Image_1.tif]
